# Polymer-Based Bioactive Luting Agents for Cementation of All-Ceramic Crowns: An SEM, EDX, Microleakage, Fracture Strength, and Color Stability Study

**DOI:** 10.3390/polym13234227

**Published:** 2021-12-02

**Authors:** Samer Al-Saleh, Turki W. Aboghosh, Mousa S. Hazazi, Khalid A. Binsaeed, Abdulaziz M. Almuhaisen, Huda I. Tulbah, Amal S. Al-Qahtani, Sara Shabib, Mashael Binhasan, Fahim Vohra, Tariq Abduljabbar

**Affiliations:** 1 Department of Prosthetic Dental Sciences, College of Dentistry, King Saud University, P.O. Box 21069, Riyadh 11475, Saudi Arabia; Salsaleh@ksu.edu.sa (S.A.-S.); htulba@ksu.edu.sa (H.I.T.); aalkahtany@ksu.edu.sa (A.S.A.-Q.); tajabbar@ksu.edu.sa (T.A.); 2 Department of General Dentistry, College of Dentistry, King Saud University, Riyadh 11545, Saudi Arabia; Dents435@gmail.com (T.W.A.); mo19sa96@gmail.com (M.S.H.); kayd435@gmail.com (K.A.B.); Abdulazizmac@gmail.com (A.M.A.); 3 Division of Operative Dentistry, Department of Restorative Dentistry, College of Dentistry, King Saud University, Riyadh 11545, Saudi Arabia; sashabib@ksu.edu.sa (S.S.); mbinhasan@ksu.edu.sa (M.B.)

**Keywords:** polymeric bioactive, ACTIVA, micro-CT, leakage, cement

## Abstract

The aim of the study was to compare microleakage and fracture loads of all ceramic crowns luted with conventional polymer resins and polymeric bioactive cements and to assess the color stability of polymeric bioactive cements. Seventy-five extracted premolar teeth were tested for fracture loads and microleakage in all-ceramic crowns cemented with two types of polymeric bioactive cements and resin cements. In addition, the degree of color change for each cement with coffee was assessed. Thirty maxillary premolar teeth for fracture loads and thirty mandibular premolar teeth for microleakage were prepared; standardized teeth preparations were performed by a single experienced operator. All prepared specimens were randomly distributed to three groups (n = 20) based on the type of cement, Group 1: resin cement (Multilink N); Group 2: polymeric bioactive cement (ACTIVA); Group 3: polymeric bioactive cement (Ceramir). The cementation procedures for all cements (Multilink, ACTIVA, and Ceramir) were performed according to the manufacturers’ instructions. All specimens were aged using thermocycling for 30,000 cycles (5–55 °C, dwell time 30 s). These specimens were tested using the universal testing machine for fracture strength and with a micro-CT for microleakage. For the color stability evaluation, the cement specimens were immersed in coffee and evaluated with a spectrometer. Results: The highest and lowest means for fracture loads were observed in resin cements (49.5 ± 8.85) and Ceramir (39.8 ± 9.16), respectively. Ceramir (2.563 ± 0.71) showed the highest microleakage compared to resin (0.70 ± 0.75) and ACTIVA (0.61 ± 0.56). ACTIVA cements showed comparable fracture loads, microleakage, and stain resistance compared to resin cements.

## 1. Introduction

Metal ceramic restorations are widely used for the rehabilitation of lost or missing teeth and have demonstrated clinical success in the last 50 years [1]. The porcelain covering the metal seems aesthetically pleasing, and the metal adds the mechanical strength. However, the underlying metal also contributes to the opaqueness of the final restoration and leaves a dark oxide line shadow in the adjacent soft tissue. The reduced light transmission and corrosion cause a tattoo in the surrounding tissues, and the abrasiveness of porcelain can damage the opposing arch [2,3].

Currently, all-ceramic restorations are considered ideal for aesthetic replacements of anterior teeth due to high translucency, adhesive bonding, and improved mechanical strength [4]. All-ceramic restorations are multilayered and composed of an inner core and an outer aesthetic fluorapatite for fracture resistance and durability, stability of color and translucency, and to effectively shape the stress profiles within the restorations [4]. However, associated complications of all ceramic restorations include the risk of bond failure mainly within the material compared to the interface of cement and restoration, occlusal fractures and ceramic chipping, staining, plaque accumulation, sensitivity, and secondary caries [5]. Therefore, for optimal properties and performance of all ceramic crowns, a robust adhesive bond of ceramic to the tooth dentin in the presence of luting agents is critical for the clinical prevention of adhesive bond failure, microleakage, fracture resistance, and aesthetic stability [6].

Conventionally, glass ionomer cements were recognized as a standard luting agent due to the high solubility that support increased fluoride release and the polymeric bioactive nature [7]. However, the low mechanical resistance raises the risk for bond failure at the interface contributing to restoration damage and failure [8]. By contrast, polymeric resin luting agents based on bis-acryl or dimethacrylate monomers show superior translucency, controlled setting time, low cement film thickness, resistance to solubility, and mechanical strength [9]. However, polymeric resin cements have their own set of associated disadvantages, which include moisture sensitivity, polymerization shrinkage and dimensional change, low bacterial resistance, and lack of dentin remineralization potential [9]. Therefore, many authors discourage the use of acid–base cements for ceramic cementations. In addition, there is a need for developing cements with organic dentin activity to allow for the repair and regeneration of biological tooth tissue (bioactivity) due to decay and caries.

Recent research has focused on developing regenerating biomaterials promoting cell activation and redevelopment of tissues. To promote the recovery of biological functions, biomimetic materials necessitate a polymeric–ceramic nanoscale support, in which the ceramic is comprised of polymeric bioactive glass containing silicate (SiO_2_), calcium oxide (CaO), and phosphorus oxide (P_2_O_5_) [10]. The different nanocrystallites from the polymeric composite, adjacent to the tissue collagen molecules trigger calcium phosphate as a precursor for dentin remineralization [11]. In addition, polymers with repeated electrolyte groups called polyelectrolytes, dissociate their electrolytes for producing charged electrolyte chains. The contemporary polymer, poly(vinylphosphonic acid) (PVPA), is a polyelectrolyte used as an analog for phosphoproteins like dentin matrix acidic phosphoprotein, critical in determining the structure, stability, and interactions of various molecular assemblies for dentin repair [12].

Conventional polymeric resin-based luting agents are not bioactive and do not actively interact with dentin and allow for molecular exchanges as they age [13,14]. Authors have proposed the use of polymeric bioactive cements, which upon interaction with moisture release ions and recharge restorative material constituents, lowering the bacterial microleakage and enhancing the marginal integrity [13,14]. ACTIVA is a contemporary polymeric bioactive agent composed of silica glass particles and an ionic-based polymeric matrix with calcium, phosphate, and fluoride ions [14]. Its superior efficacy and reactivity facilitate the benefits of improved durability and antimicrobial resistance; the chemical bond with dentin reduces the dentinal sensitivity and microleakage [15].

Studies performed recently using micro-CT showed a significant difference in the microleakage between the resin-based cement and novel polymeric bioactive cement [15,16]. However, few studies denied any such difference when performed in class V cavities [17,18]. A recent study by Vohra et al. compared the bond strength of resin and bioactive cements for ceramic crowns, showing low microleakage and comparable dentin bond strength of bioactive to resin cement [19]. However, studies comparing fracture strength, color stability, and microleakage of different bioactive cements are not available. Moreover, research data relevant to polymeric bioactive cement performance for luting all-ceramic dentin-bonded crowns is limited. Therefore, the study aimed to assess polymeric bioactive cements in comparison to resin cements for restorative microleakage, crown fracture loads, and color stability with crown specimens.

## 2. Materials and Methods

Variables that were tested in this study include fracture loads and microleakage in dentin-bonded all-ceramic crowns cemented with two types of polymeric bioactive cements and a resin-based cement. In addition, the degree of color stability of polymeric bioactive and resin cements was assessed. The testing and reporting for the experiments were performed according to the “checklist for performing in vitro studies (CRIS guidelines)”.

Seventy-five extracted premolar teeth were collected after orthodontic extractions fulfilling the inclusion criteria (sound crowns, roots, and similar size dimensions). Teeth were sterilized and stored in a 0.1% solution of thymol solution. Teeth were aligned vertically using a surveyor and mounted using orthodontic acrylic resin 2 mm below the cement–enamel junction (CEJ) in polyvinyl cross-sectional rings.

### 2.1. Chemical Analysis of Cements

To evaluate the elemental distribution in the cements (Multi-link, Ceramir, and ACTIVA) the scanning electron microscopy (SEM) and Energy Dispersive X-ray (EDX) techniques were employed [20]. Cements were placed on the aluminum stubs and sputter coated with a layer of gold in a sputter coater machine (Baltec sputter, Scotia, NY, USA). SEM micrographs were then obtained at different magnifications with an accelerating voltage of 10 kV in an SEM machine (FEI Quanta 250, Scanning Electron Microscope, Thermo Fisher Scientific, Hillsboro, OR, USA).

### 2.2. Specimen Preparation

Thirty maxillary premolar teeth for fracture strength and thirty mandibular premolar teeth for microleakage were prepared; standardized teeth preparations were performed by a single experienced operator (FV). The dimensions of dentin-bonded crown tooth preparations were 1 mm axially, 1.5 mm occlusal (occluso-gingival = 3 mm, mesio-distal = 2 mm, bucco-lingual = 4 mm), 1 mm heavy chamfer (0.5 mm above the CEJ), and a 16 degree total convergence angle. Tooth preparations were standardized using putty indices and a periodontal probe. All prepared specimens were randomly distributed to three groups (n = 20) based on the type of cement:

Group 1: resin cement (Multilink N, Ivocalr Vivadent, Buffalo, NY, USA);
Group 2: polymeric bioactive cement (ACTIVA polymeric bioactive cement, Pulpdent, Watertown, MA, USA);
Group 3: polymeric bioactive cement (Ceramir C&B, Daxo dental, Uppsalla, Sweden).

For the crown fabrications, impressions of individual tooth preparations were recorded using small paper cups and a combination of light body and regular body polyvinyl siloxane (PVS) impression materials (Express Impression Material, 3M ESPE, St. Paul, MN, USA). The die stone fabrications were followed by wax-ups for each specimen with standardized dimensions (1 mm axially, 2 mm from the cusp tip, and 1.5 mm from the occlusal fissure). IPS EMAX (EMAX, Ivocalr Vivadent, Buffalo, NY, USA) ceramic ingots were used to fabricate the pressed ceramic crowns by lost wax technique and hot pressing. An experienced laboratory technician performed all laboratory procedures. All crowns in each group were etched with 9.5% HF acid (HF acid, Ceram-Etch Gel, Gresco products, Stafford, TX, USA) for 30 s followed by surface cleaning in an ultrasonic bath and distilled water (5 min). A silane coupling agent was applied to dried and cleaned ceramic surfaces (Monobond S, Ivoclar Vivadent, Buffalo, NY, USA) and allowed to dry for 5 min. All crown specimens were divided into their respective cement groups prior to cementation.

The cementation procedures for all cements (Multilink, ACTIVA, and Ceramir) were performed according to manufacturers’ instructions. Tooth dentin was dried with cotton pellets to remove external moisture without desiccation. Cement was dispensed with an automix syringe into the crown fitting surface and seated onto the prepared tooth under a static load of 20 N for 2 min. The excess was removed with a plastic instrument and a microbrush, and photopolymerization (Bluephase, Ivoclar, Vivadent, Buffalo, NY, USA) was performed for 20 s at each surface (occlusal, buccal, lingual, mesial, distal), with a total duration of 100 s. All specimens were aged using thermocycling for 30,000 cycles (5–55 °C, dwell time 30 s) (Sensoquest GmbH, Göttingen, Germany). Prior to the specimen testing, the tooth–crown complex was stored in a humid oven at 37 °C (incubator, Memmert Universal Oven, Memmert, Schwabach, Germany).

For the staining and degree of color change assessment, 15 extracted maxillary premolars were sectioned 2 mm below the cement–enamel junction with a diamond bur. Teeth were mounted in polyvinyl cross-sections using orthodontic acrylic resin (Caulk Orthodontic Resin, Dentsply Caulk, Milford, DE, USA). Standardized cavity preparations were performed using a mounted high speed dental handpiece and a diamond donut bur on the buccal and lingual tooth surfaces of each tooth resulting in 10 restorations in each cement group (n = 10). The burs were inserted perpendicular to the tooth surface to prepare similar round cavities (diameter = 3 mm, depth = 1.5 mm). Teeth specimens were randomly divided into three groups based on cement type:

Group A: resin cement (Multilink N, Ivocalr Vivadent, Buffalo, NY, USA);
Group B: polymeric bioactive cement (ACTIVA polymeric bioactive cement, Pulpdent, Watertown, MA, USA);
Group C: polymeric bioactive cement (Ceramir C&B, Daxo dental, Uppsalla, Sweden).

Restoration protocols for staining assessment were performed according to manufacturers’ instructions. For resin cements (Group A), tooth dentin was etched with 34% phosphoric acid (EZ Etch, Dentsply Sirona, Charlotte, NC, USA) for 15 s and washed and dried for 20 s. A bonding agent (Prime & Bond NT, Dentsply, Charlotte, NC, USA) was applied for 20 s and photopolymerized (Bluephase, Ivoclar, Vivadent) for 10 s. For polymeric bioactive cements, (Group B: ACTIVA and Group C: Ceramir), the dentin was dried with cotton pellets to remove external moisture without desiccation. For all specimens, equal amounts of cement were placed into the prepared cavities using an automix syringe dispenser and the excess cement was removed using a plastic instrument and a microbrush. Photopolymerization of the restoration was performed (Bluephase, Ivoclar, Vivadent) for 20 s each through the gingival, occlusal, mesial, and distal surfaces (total exposure of 80 s).

### 2.3. Failure Load Testing

All cemented specimen crowns were tested for fracture strength under standard loads using a universal testing machine (INSTRON 5965, Norwood MA USA). Specimens were secured in the load cell with a customized jig and a constant load was applied on the center of occlusal through a 3 mm diameter round head. Loads were applied at a crosshead speed of 1 mm/min until the fracture of the crown. The fracture strength values were obtained in megapascals (MPa).

### 2.4. Microleakage Assessment

The root surfaces of all crown-bonded specimens were painted with two layers of nail varnish. Specimens were immersed in 50% silver nitrate (AgNO3) in the dark for 12 h followed by washing (water). Specimens were further placed in a photo-developing solution under a fluorescent light for 12 h. For the assessment of microleakage, bonded specimens were securely placed in a micro-CT chamber (Bruker SkyScan 1173 Kontich, Belgium). Scanning configuration included 86 kV voltage, 93 μA anode current, 620 ms exposure time, isotropic resolution of 16 μm image pixel size, brass filter, 0.25 rotation step for 360° angle, frame averaging of four for improved signal to noise ratio, and random movement of eight to minimize ring artifacts. The projected images were reconstructed using N-Recon^®^ program version 1.6.1.3 (Bruker Skyscan, Kontich, Belgium) to produce cross-sections of images. Images were saved in a 16 bit TIF file format. Reconstructed images were loaded in the Dataviewer^®^ program (Bruker Skyscan, Kontich, Belgium) software to determine accurate positioning and visual inspection. The volume of silver nitrate penetration was quantified with the CTAn^®^ program (Bruker Skyscan, Kontich, Belgium) by selecting a binarized threshold value corresponding to the amount (silver nitrate).

### 2.5. Staining Assessment

The restored tooth specimens were assessed for color change after immersion in the coffee solution. The color of the specimens was initially assessed after 24 h of restoration as a baseline measurement (control). A resin jig was used to reproducibly place each specimen in the ColorEye 7000A Spectrophotometer (Grand Rapids, MI, USA). Prior to the color measurements, the spectrophotometer was calibrated according to the National Institute of Standards and Technology (NIST) tiles. The setting parameters of the spectrophotometer were 10 nm of wavelength interval, spectral range of 360 to 750 nm, and a 45° reflectance angle. All specimens were cleaned with ethyl alcohol absorbent paper prior to the color measurements. The background for all measurements was black. Three color measurements were performed at room temperature for each specimen, and an average was calculated for each sample.

For specimens, L *, a *, and b *, the axes were identified using the CIELAB color space, where “L ” is brightness to darkness ranging from 0 to 100, the “a ” axis represents red to green with coordinate values ranging from 90 to 70, and the “b ” axis represents yellow to blue with coordinates ranging in value from −80 to 100. Initial assessments were performed for 10 restorations in each cement group.

Ten samples in each group were immersed in coffee (instant black, Nescafe, Switzerland) solutions. Five grams of coffee crystals were mixed with 350 mL of hot water at 100 °C. This was followed by filtration after 1 min. Specimens were immersed in coffee for 2 weeks, and the coffee solutions were replaced every 24 h. Post-immersion, the specimens were washed with running water (5 min) and dried with absorbent paper. Each specimen was assessed for color coordinates L, a, and b with a Color Eye 7000A Spectrophotometer, as presented in the earlier text. The differences in color were identified by calculating the change in coordinates (ΔL, Δa, and Δb values) [21]. The overall CIELAB color difference values for each specimen (ΔE) between baseline and post-immersion color were calculated based on the formula adopted from the CIE technical report on colorimetry.

ΔE = [(ΔL)^2^ + (Δa)^2^ + (Δb)^2^]1/2 (1)

The distribution of data was assessed using the Kolmogorov–Smirnov test. Analysis of variance (ANOVA) and the Tukey Kramer multiple comparisons test were employed to compare groups for fracture strength and microleakage and color change.

## 3. Results

**Figure 1:**
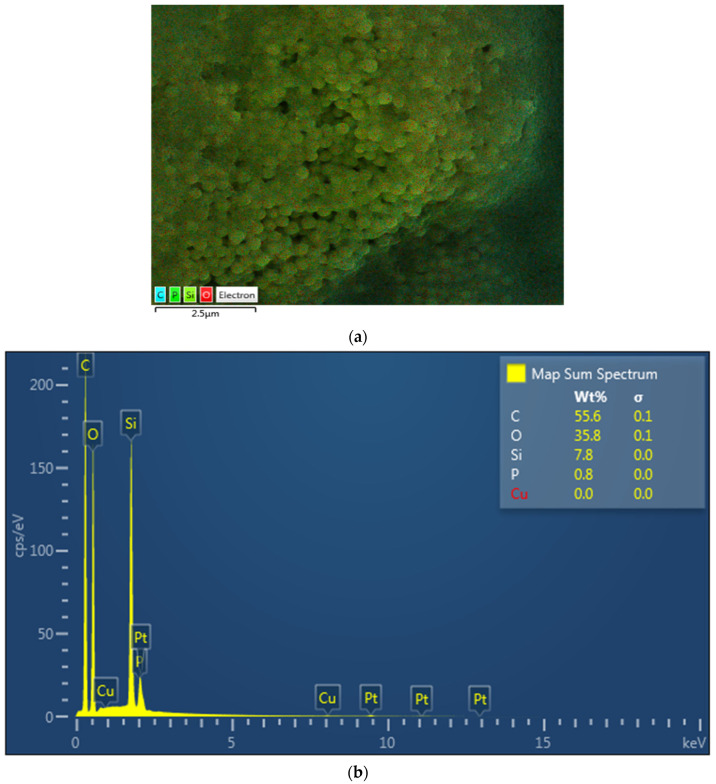
(**a**): Electron image of the resin cement, Multilink. (**b**): EDX for Multilink cement showing Si for glass silica, O for spheroid oxides, and C for carbon in the dimethacrylate and HEMA monomers.

**Figure 2:**
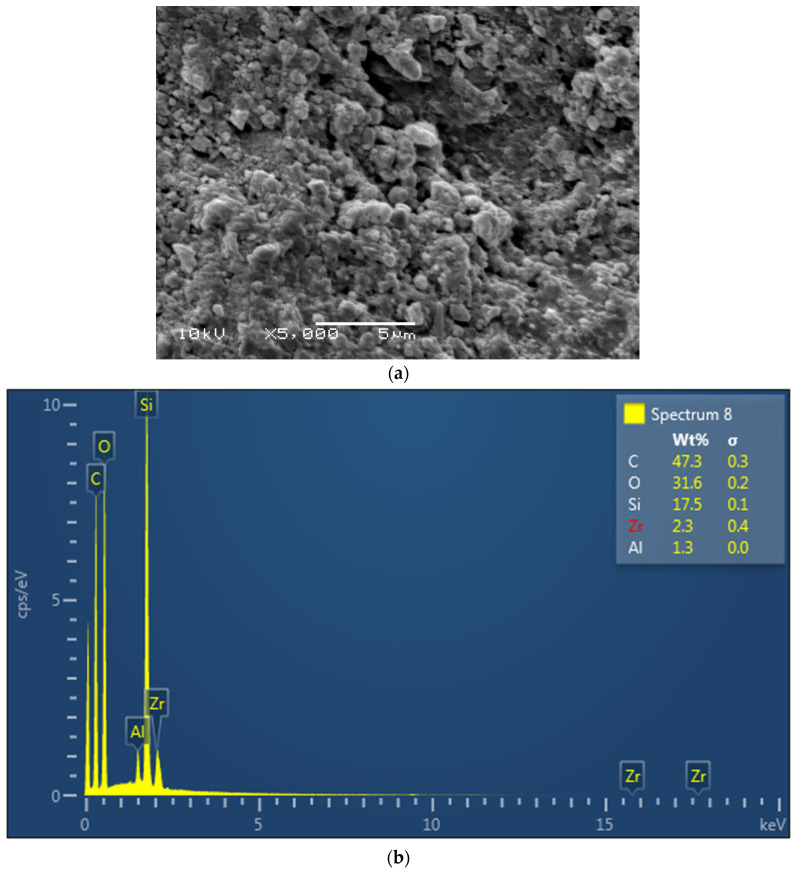
(**a**): Electron image of the bioactive cement, Ceramir. (**b**)**:** EDX for Ceramir showing Al, Si, and O from the aluminate (Al) and glass (Si-Zr) powder in the filler and other oxides (O).

**Figure 3:**
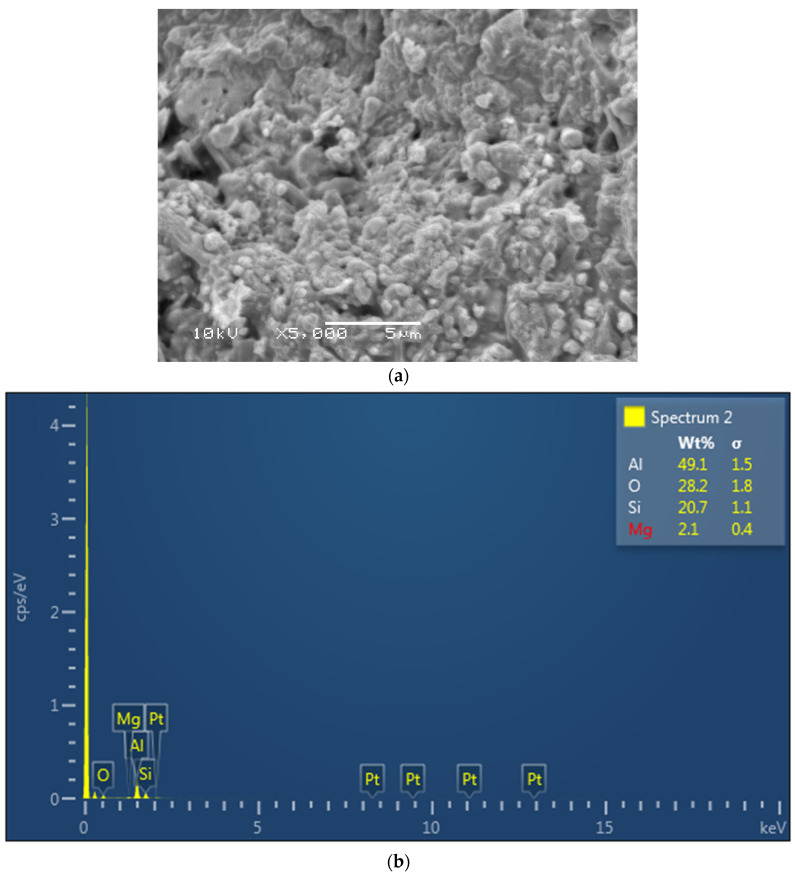
(**a**): Electron image of the bioactive cement, ACTIVA. (**b**):EDX for ACTIVA showing alumina, Si, and O from the aluminosilicate glass powder in the filler.

The EDX mapping revealed the presence of Si for glass, spheroid oxides (O), and carbon (C) in the dimethacrylate and HEMA monomers in resin cement (Multilink) (Figure 1a,b). The Ceramir cement revealed the presence of Al, Si, and O from the aluminate (Al) and glass (Si-Zr) powder in the filler and other oxides (O) in the cement (Figure 2a,b). Lastly, EDX analysis of ACTIVA showed alumina, Si, and O from the aluminosilicate glass powder in the filler (Figure 3a,b).

**Figure 4:**
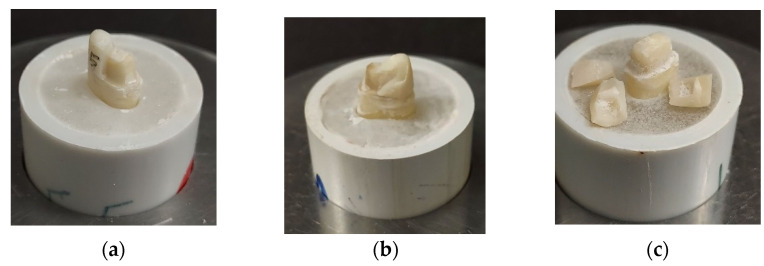
Fractured specimens after failure load assessments in study groups (**a**) Multilink (**b**) ACTIVA (**c**) Ceramir.

The present study assessed the restorative microleakage and fracture loads of restorative crowns luted with Ceramir, polymeric bioactive and resin cements, and color stability. The highest mean for the fracture loads were observed in Group 1 (49.5 ± 8.85) whereas the lowest mean was measured in the Group 3 (39.8 ± 9.16) cement (Table 1). ANOVA presented a significant difference among the study groups for failure loads (*p* < 0.01). However, Group 2 showed a comparable failure load mean to Group 1 (resin cement) (48.7 ± 6.59). Furthermore, Group 1 showed a greater range for fracture loads compared to the Group 2 specimens (ACTIVA). Figure 4 presents the failed crown specimen, post fracture testing of Group 1 (a), Group 2 (b), and Group 3 (c) specimens.

**Figure 5:**
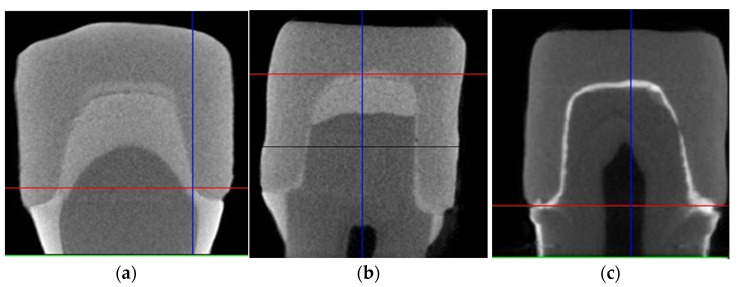
Micro-CT images of microleakage assessments among (**a**) Group 1-resin cement, (**b**) Group 2-ACTIVA, and (**c**) Group 3-Ceramir.

Crown fractures are related to the amount of microleakage. Table 2 presents means and standard deviations for microleakage among different cement groups. The highest amount of microleakage was observed in Group C-Ceramir (2.563 ± 0.71) compared to Group A-resin (0.70 ± 0.75), and Group B-ACTIVA (0.61 ± 0.56). Although a significant difference was appreciated among the three cement groups, nevertheless, Group A (resin) and Group B (ACTIVA) presented with comparable (*p* > 0.05) and low microleakage values. The micro-CT scans highlighting the level of microleakage in each cement group are shown in Figure 2. In Group C, (Figure 5c) the prominent leakage radiant line is appreciated, whereas in Group A (Figure 5a) and Group B (Figure 5b), the presence of radiopaque silver nitrate is minimal. The boundary of the crown near the tooth neck is more evident in Group A (resin) compared to group B (ACTIVA).

Prolonged risk of microleakage is also a significant contributor to change in color. Color stability, i.e., the change in color (ΔE) was significantly different among the study groups (*p* = 0.02) (Table 3). Ceramir (Group C) demonstrated the highest susceptibility to change in color (18.84 ± 5.42 ΔE) followed by resin luting cement (Group A) (11.43 ± 3.54 ΔE) and ACTIVA (Group B) (5.79 ± 6.24 ΔE), respectively. Individual comparisons among the groups showed the significantly higher color change in Group C compared to Groups A and B (*p* < 0.05). In addition, Group B specimens showed significantly lower color change (good color stability) compared to specimens in Groups A and C (*p* < 0.05).

## 4. Discussion

The present study investigates the fracture strength and microleakage of all-ceramic crowns luted with polymeric bioactive and resin cements along with cement color stability. The perceived outcome displayed a significant difference in fracture loads, microleakage, and color stability among the study groups. Both polymeric bioactive cements (ACTIVA and Ceramir) showed significant differences in properties, and the hypothesis was partly rejected. The rationale for these findings is manifold, which includes material composition, cement interaction with the dentinal matrix, and impact of the dissolution of restoration.

The present study pointed out an evident difference of microleakage within two polymeric bioactive cement groups compared to the standard resin cement. By definition, microleakage occurs at the point where there is a marginal nanogap caused by polymerization shrinkage, microcracks, differences in the thermal expansion coefficient between the restoration and the tooth structure, layering, and inadequate finishing and polishing [22,23]. However, any restoration present in the oral cavity is constantly subject to saliva that increases the risk of bacterial ingression leading to early bond failure [18]. Similarly, previous studies demonstrate the volumetric leakage between composite restoration and crown using a radioactive tracer [24,25]. Polymeric bioactive cements showed the least amount of microleakage compared to resin cements due to dentin interaction. In ACTIVA cements, the presence of moisture initiates the ionization process leading to the exchange of calcium and phosphate ions to form a hydroxyapatite complex creating a tight seal [22,24]. The available calcium and phosphate ions react with the residual apatite of the dentin surface, causing nucleation and growth of nano-apatite crystals in the internal compartments of dentin collagen [26,27]. In addition, it is suggested that the availability of polymeric electrolytes in polymeric bioactive cements such as dentin matrix acidic phosphoprotein regulate interactions of molecules involved in remineralization [12]. This, along with other processes in polymeric bioactive cements, complement the findings of the present study.

The material composition has been shown to influence the material strength and clinical properties. In the present study, ACTIVA (rubberized polymeric bioactive) cements showed comparable fracture loads and microleakage to resin; however, ACTIVA also showed higher fracture loads and lower microleakage than the other bioactive cements investigated in the study (Ceramir). The rubberized polymer in ACTIVA cements enhance the modulus of elasticity of the material, hence, showing fracture strength higher than Ceramir and comparable to resin cements in the present study. This can be attributed to variability in material composition. In addition, through EDX analysis in the study, alumina, Si, and O were observed based on the aluminosilicate glass powder in the filler of ACTIVA, possibly contributing to the positive cement outcomes. Many authors provided evidence pointing out that the resin matrix (Multilink) provides internal strength to the restorative material complementing fracture strength with a good seal [28,29]. EDX analysis of the resin cements showed the presence of Si for glass, spheroid oxides (O), and carbon (C) in the dimethacrylate and HEMA monomers in the present study, which is in line with the cement composition. Ceramir cements are a hybrid material based on calcium aluminate and ceramic ionomers, with greater susceptibility to dissolution compared to ACTIVA that has an ionic resin matrix [27]. For Ceramir, EDX analysis revealed the presence of Al (calcium aluminate), Si and Zr (glass powder filler), and other oxides (O) in the cement. The nanofiller in the polymeric resin matrix increases the surface for interaction and cement setting, which augments the material strength with greater monomer conversion and ionic release [30,31]. In addition, the changing pH of the oral cavity also favors the revitalization of the tooth dentin and enhances the bond strength by producing a seal at the dentin cement complex [32,33]. Henceforth, ACTIVA showed higher fracture strength compared to Ceramir indicating the impact of composition on fracture strength and microleakage.

In the present study, a significant difference was observed within the study groups in terms of color change, especially between the two polymeric bioactive cements. According to Vohra et al. [19], resin cements produce a greater level of microleakage in the absence of hydroxyethylmethacrylate (HEMA) in the adhesive dentin polymer, which contributes to cement discoloration. The continuous microleakage leads to peptides in the dentin collagen reacting with hydrogen, resulting in a complex susceptible to collapse and, thus, compromising the seal and bond integrity. A similar pattern of material dissolution and leaching was observed in the Ceramir cement, as it is composed of glass ions. This eventually results in the breakdown of the seal and discoloration of the cement. However, polymeric bioactive materials demonstrated the unique property of interlocking and binding with a dentinal complex, which further strengthens the dentin bond [22,30]. Acid etching tends to open up dentinal tubules allowing a greater degree of material penetration and reducing susceptibility to microleakage and color change [34,35]. Nevertheless, in the present study, the outcomes for resin and polymeric bioactive cements were similar and better compared to the Ceramir cement, which presented greater susceptibility to color change. It is pertinent to mention that these polymeric bioactive materials show bioactivity and remineralization, and minerals are incorporated in the dentin surface, but its polymer matrix interaction is different from physiological recovery [36]. The complete recovery of properties of exposed and demineralized dentin due to polymeric bioactive agents needs the formation of intra- and extrafibrillar minerals between the collagen fibers. Therefore, further advances in the polymeric bioactive material development are pivotal.

From a clinical perspective, the study suggests that polymeric bioactive cements (ACTIVA) have the potential to perform similarly to polymer-based resin cements with regards to ceramic crown failure, microleakage resistance, and color stability (stain resistance). To replicate oral conditions, the specimens were aged through thermocycling, and ageing is known to compromise mechanical properties of the tested cements [37,38]. However, the thermocycling protocol cannot be standardized in an in vitro setting. In addition, intraoral stresses are dynamic, continuous, repetitive, and short-lived; however, in vitro fracture testing employed in the present study used static loads of high magnitude until fracture. Moreover, apart from material ageing, the dynamics of oral conditions and tooth structure also vary from person to person, which greatly influence the degree of microleakage. Furthermore, microleakage assessments using a silver nitrate particle tracer and micro-CT can be compromised by the sliver particle size and reconstruction of images, leading to false negatives. Therefore, to infer further clinical applications of polymeric bioactive cements in all ceramic crown performance and function, further randomized controlled trials evaluating polymeric bioactive cements and ceramic crown durability are recommended.

## 5. Conclusions

ACTIVA (polymeric bioactive cement) demonstrated comparable fracture loads, microleakage, and stain resistance compared to resin cements. Ceramir presented poor fracture resistance, color stability, and microleakage in comparison to ACTIVA and resin cements. Polymeric bioactive cement (ACTIVA) as a luting agent has the potential for adequate clinical performance and durability as conventional polymeric resin cements for all ceramic crowns.

## Figures and Tables

**Table 1:** Means and standard deviations (SD) for failure loads (MPa) among study groups.

Type of Cement	Mean (MPa)	SD	Maximum	Minimum	*p* Value *
Group 1-Resin	49.5 ^A^	8.85	58.0	41.72	<0.01
Group 2-ACTIVA	48.7 ^A^	6.59	54.6	41.7	
Group 3-Ceramir	39.8 ^B^	9.16	50.07	29.42	

Megapascals (MPa). Different superscript uppercase alphabets in column denote statistically significant differences (*p* < 0.05) (Tukey post hoc test) * ANOVA.

**Table 2:** Mean and standard deviation values for microleakage among different cement groups.

Type of Cement	Mean & SD	*p* Value *
Group 1-resin	0.70 ± 0.75 ^A^	0.00098
Group 2-ACTIVA	0.61 ± 0.56 ^A^	
Group 3-Ceramir C&B	2.563 ± 0.71 ^B^	

* ANOVA. Different superscript uppercase alphabets in column denote statistically significant differences (*p* < 0.05) (Tukey post hoc test).

**Table 3:** Means and SDs for ΔE values among the study groups.

Study Groups	Mean & SD	*p* Value *
Group A-resin	11.43 ± 3.54 ^A^	0.0021
Group B-ACTIVA	5.79 ± 6.24 ^B^	
Group C-Ceramir C&B	18.84 ± 5.42 ^C^	

* ANOVA. Different superscript uppercase alphabets in column denote statistically significant differences (*p* < 0.05) (Tukey post hoc test).

## Data Availability

Data of the study is available on request from the corresponding authors.
